# Prevalence of markers of celiac disease in Colombian children with diabetes mellitus type 1

**DOI:** 10.25100/cm.v49i3.3650

**Published:** 2018-12-30

**Authors:** Carlos Alberto Velasco-Benítez, Ángeles Ruíz-Extremera, Audrey Mary Matallana-Rhoades, Sandra Carolina Giraldo-Lora, Claudia Jimena Ortíz-Rivera

**Affiliations:** 1 Universidad del Valle, Escuela de Medicina , Departamento de Pediatría. Cali, Colombia; 2 Universidad de Granada, Estudiante de doctorado en Medicina Clínica y Salud Pública. Granada, España.; 3 Universidad de Granada. Granada, España; 4 Hospital Universitario del Valle “Evaristo García”, Endocrinología Pediátrica. Cali, Colombia

**Keywords:** Prevalence, genotype, celiac disease, potential celiac disease, type 1 diabetes mellitus, children, HLA-DQ antigens, Transglutaminases, prolamins, glutens, Prevalencia, genotipo, enfermedad celíaca, diabetes mellitus tipo 1, niños, antígeno HLA-DQ, Transglutaminasas, prolaminas, gluten

## Abstract

**Introduction:**

Although the association between diabetes mellitus type 1 (T1DM) and celiac disease (CD) is well established; there are only a few studies that focus on South American children, haplotypes and their possible associations.

**Objective:**

To determine the prevalence of CD markers in a group of children with T1DM and to analyze the associated clinical, immunological and genetic manifestations.

**Methods:**

A prevalence study focusing on children with T1DM who were assessed based on variables including sociodemographics, anthropometric information, disease characteristics, laboratory results and family medical history. In partitipants a positive tTG2 (Ig A anti-transglutaminase), a duodenal biopsy and genotype were performed. The proportion of children with T1DM and CD was estimated (CI 95%). Determinations of central tendency, univariate and bivariate analysis, were also performed; *p* <0.05 was considered significant.

**Results:**

Thirteen (8.4%) of the 155 children (53.6% girls, 11.0 ±3.6 years, 2-18 years) with T1DM were tTG2 positive, four had CD (2.6%), seven had potential CD (4.5%) and nine were HLA DQ2/DQ8 positive (5.8%). Children with T1DM and CD had their last ketoacidotic episode (21.5 ±30.4 months versus 69.5 ±38.8 months, *p*= 0.0260) earlier than children with T1DM and potential CD. There were no differences with anthropometry or with the laboratory results regarding glycemic control.

**Conclusions:**

The prevalence of CD in these children with T1DM is higher than that reported in other South American countries. The prevalence of CD was found to be associated with the time of presentation of T1DM and its main allele, the DQ2/DQ8. These findings are different from what has been described in other places around the world.

## Resumen

**Introducción:**

A pesar que la asociación entre diabetes mellitus tipo 1 (DMT1) y enfermedad celíaca (EC) está bien establecida; hay pocos estudios en niños suramericanos sobre haplotipos y sus posibles asociaciones.

**Objetivo:**

Determinar la prevalencia de marcadores de EC en un grupo de niños con DMT1, analizando las manifestaciones clínicas, inmunológicas y genéticas.

**Métodos:**

Estudio de prevalencia en niños con DMT1 a quienes se les tomaron variables sociodemográficas, antropométricas, de la enfermedad, paraclínicas y familiares metabólicas. A los niños con IgA anti-transglutaminasa (tTG2) positivos, se les realizó biopsia duodenal y genotipo. Se estimó la proporción de niños con DMT1 y EC y su IC 95%; medidas de tendencia central, análisis univariado y bivariado, siendo significativa una p <0.05.

**Resultados:**

Trece (8.4%) de los 155 niños (53.6% niñas, de 11.0 ±3.6 años, 2-18 años) con DMT1 fueron tTG2 positivos, cuatro presentaron EC (2.6%), siete EC potencial (4.5%) y nueve HLA DQ2/DQ8 (5.8%). Los niños con DMT1 y EC presentaron más pronto su último episodio cetoacidótico (21.5 ±30.4 meses versus 69.5 ±38.8 meses, *p*= 0.0260) que los niños con DMT1 y EC potencial. No hubo diferencias con la antropometría ni con los paraclínicos del control glicémico.

**Conclusiones:**

La prevalencia de EC en estos niños con DMT1 es superior a la de otros países suramericanos; estando asociada al tiempo de presentación de la DMT1 y su principal alelo el DQ2/DQ8, hallazgos diferentes a lo descrito a nivel mundial.

## Introduction

Celiac disease (CD) is a systemic disorder mediated by the immune system and caused by gluten and related prolamins, it is found in genetically susceptible individuals and is characterized by the presence of a variable combination of gluten-dependent clinical manifestations, CD-specific antibodies, HLA-DQ2 or HLA-DQ8 haplotypes and enteropathy ^1^.

The association between type 1 diabetes mellitus (T1DM) and CD is well established; however, in recent years, several studies have shown that the prevalence of CD in diabetic patients is even higher than previously thought ^2^.

The guidelines for the diagnosis of CD from the European Society for Paediatric Gastroenterology, Hepatology and Nutrition (ESPGHAN) suggest testing asymptomatic children for CD when they have an increased genetic risk for developing it, as is seen in patients with type 1 diabetes mellitus (T1DM) ^1^.

The highest prevalence reported worldwide of CD is in the Sahara Desert, Africa (5.6%), followed by Oceania (1.2%), Europe (1.0%), the United States (0.8%), Asia (0.3% to 0.7%), and Brazil and Argentina (0.1% to 0.6%) ^3^. This prevalence is higher in patients with T1DM (2.4% to 16.4%) ^4^. In a systematic and meta-regression review conducted in Colombia, it is concluded that CD seems to be a rare condition among Colombians ^5^. It is well known worldwide that there is a higher prevalence of CD in children with T1DM compared to the general population ^6^, and that most of these children are asymptomatic at the time of diagnosis ^7^ ^,^ ^8^. However, it has yet to be established in Colombia whether this risk is greater in children under 5 years old (as has been demonstrated by the Europeans) ^9^, whether there is a predominance of the female gender to develop CD (as there is in the general adult population) ^10^, and whether there is a relationship among CD, T1DM and glycemic control (as is still controversial) ^11^ ^-^ ^14^. Additionally, there have been no previous Colombian studies about the prevalence rates, clinical characteristics and laboratory results in children with coexisting CD and T1DM. Therefore, the databases of the Pediatric Endocrinology Service of the Hospital Universitario del Valle "Evaristo García" in Cali, Colombia were analyzed. The data of children diagnosed with T1DM were analyzed in order to determine the prevalence of CD markers in a group of children with T1DM. Clinical, immunological and genetic manifestations were included in the analysis.

## Materials and Methods

T1DM was diagnosed in participants when there was evidence of beta cell destruction in children older than 6 months of age, regardless of whether they presented with ketoacidosis, and whether they had other autoimmune diseases. T1DM was diagnosed in participants older than 10 years of age if they were obese, in the same way, until there is evidence of absence of autoimmunity ^15^. CD was diagnosed when anti-transglutaminase IgA (tTG2) was positive, the HLA DQ2 and HLA DQ8 haplotypes were compatible, and when duodenum histology showed intestinal villus abnormalities with Marsh grade II or higher in the setting of gluten-dependent clinical manifestations. Potential CD was diagnosed in the absence of histological abnormalities on the duodenal biopsy when the tTG2 was positive and the HLA haplotypes were compatible, with or without signs and symptoms ^1^.

An observational prevalence study was performed in children diagnosed with T1DM who presented between 2 August 2013 and 23 February 2017 at the Pediatric Endocrinology Service of the Hospital Universitario del Valle "Evaristo García" in Cali, Colombia. This hospital is a third-level care institution located in the southwest of the country. The inclusion criteria were children diagnosed with T1DM, both male and female; age older than 6 months of age; and previous consumption of gluten in their complementary diet. 

The exclusion criteria were children diagnosed with congenital diabetes mellitus, being in a ketoacidotic coma, the presence of associated chromosomal abnormalities such as Down syndrome, the presence of other associated autoimmune diseases such as hypothyroidism, a previous diagnosis of CD, and the presence of inflammatory bowel disease. 

Sociodemographic variables that were considered included age, sex, race and origin. Anthropometric variables included weight and height. The study also considered the length of time since being diagnosed with T1DM, the number of ketoacidotic comas and the date of the last ketoacidotic coma. Laboratory results, such as glycosylated hemoglobin, hemoglobin and glycemia were recorded, and the metabolic relationships between hypertension, diabetes, being overweight and obesity were considered. The digestive clinical symptoms that were studied were constipation, vomiting, abdominal distension, steatorrhea, diarrhea, abdominal pain, flatulence and weight loss. Screening was carried out using an anti-transglutaminase IgA (tTG2) Biocard ™ Celiac Test (Ani Biotech, Vantaa, Finland, 97.4% sensitivity, 96.9% specificity) ^16^ ^,^ ^17^. In the participants with a positive tTG2, a minimum of 4 biopsies of the bulb and second duodenal portion were taken. The evaluation of the biopsy material was made by the same pathologist after hematoxylin-eosin staining. Immunohistochemistry of the common leukocyte antigen was used for the evaluation of the intraepithelial leukocyte count, and the presence of more than 30 intraepithelial lymphocytes versus 100 epithelial cells detected by common leukocyte antigen was considered as intraepithelial lymphocytosis ^18^ ^,^ ^19^. According to Marsh-Oberhuber histopathology, the sample was classified as Marsh I when the results showed an "infiltrative lesion", as Marsh II when there was an "infiltrative-hyperplastic lesion", and as Marsh III when there was "hairy atrophy" (partial IIIa, subtotal IIIb and total IIIc) ^20^. These children underwent genotyping of HLA DQ2 and HLA DQ8, by polymerase chain reaction ^21^.

The parents or guardians of the participants signed informed consent forms, as did the children who were over 8 years of age. This study was approved by the Ethics Committee of the Universidad del Valle and the Hospital Universitario del Valle "Evaristo García" in Cali, Colombia.

The sample size included all children diagnosed with T1DM who met the inclusion criteria of the study, agreed to participate in the study and presented to the Pediatric Endocrinology Service of the Hospital Universitario del Valle "Evaristo García" in Cali, Colombia. Percentages, percentiles, averages, medians and other descriptive measures were estimated to a CI 95% with their corresponding standard deviations and ranges. To evaluate the possible associations, univariate analysis was performed for each of the variables. In addition, we explored the possible association between the variables of exposure of greatest interest and other covariates, and between the outcome variable of interest (CD) and the other covariates, in order to evaluate the possible existence of confusion. To do this, graphs and 2x2 tables were constructed and the ORs with their respective confidence intervals (95%) were estimated. To assess the statistical significance, the Fisher`s exact test was used and a *p* <0.05, two-tailed value, was considered statistically significant.

## Results

A total of 155 children were included (83 male), with an average age of 11 years (2 to 18) and a diagnosis of T1DM. They had an average duration of the disease of 46.1 months (0 to 180 months), with an average of one episode of ketoacidotic coma (0 to 7 episodes). The last average ketoacidotic episode was 20.5 months prior (0 to 163 months). The majority (73.6%, 114/155) of the participants presented with some family history of metabolic disorders. The laboratory results were: glycosylated hemoglobin of 9.1% (5.2 to 19.0%), glycemia of 193.1 g/dL (36.1 to 600.0 g/dL) and hemoglobin of 12.9 g/dL (9.1 to 15.0 g/dL). Most of the participants came from urban cities (n=109, 70.3%). The tTG2 was positive in 8.4% (13/155) of the cases. The anthropometric parameters are shown in Table 1.

**Table 1:** Characteristics of children with type 1 diabetes mellitus and anti-transglutaminase IgA (n = 155)

Variables	tTG2	
	Positive (n= 13)	Negative (n= 142)
Sociodemographic	8.4%. (CI 95%: 6.2 to 10.6)	91.6% (CI 95%, 89.3 to 93.8)
Age (years) X (range)	10 (5-16)	11 (2-18)
Sex (female:male)	7:6	76:66
Origin (urban:rural)	10:3	99:43
Race (white:other)	6:7	73:69
**Antecedents**	** **	** **
Family members with metabolic diseases (n, %)	10 (76.9)	104 (73.2)
**Evolution of the disease**	** **	** **
Duration (months) X (range)	38.1 (0-140)	46.8 (0-180)
No. ketoacidotic comas X (rank)	1 (0-3)	1 (0-7)
Last ketoacidotic coma (months) X (rank)	36.4 (0-97)	19.4 (0-163)
**Nutritional status according to WHO**		
Normal:Malnutrition	8:5	102:40
Normal:Altered height	13:0	126:16
**Laboratory results**	** **	** **
Hemoglobin (g/dL) X (range)	13.2 (11.0-15.8)	12.9 (9.1-15.8)
Glycosylated hemoglobin (%) X (range)	9.3 (5.6-12.0)	9.1 (5.2-9.0)
Glycemia (g/dL) X (range)	179.2 (57-560)	194.4 (36.1-600.0)

X=mean; n=number

**Figure 1:**
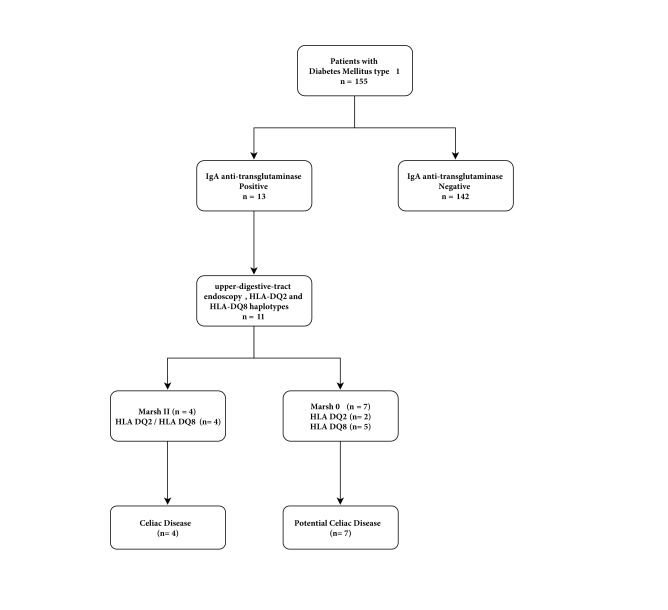
Flow chart for the study of children with T1DM and CD

Of the 155 children included, 13 had positive tTG2 (8.4%), and eleven of them underwent endoscopy and biopsy of the upper digestive tract (EUDT). Their HLA DQ2 and HLA DQ8 haplotypes were also determined (Fig. 1). The 4 children with CD as determined by positive immunohistochemistry had a number and location consistent with CD in the CD3, CD8 and CD45 antibodies compared with appropriate controls. In Table 2, the general characteristics of the 11 children with T1DM, potential CD (n= 7) and CD (n= 4) are described.

**Table 2:** General characteristics in children with type 1 diabetes mellitus and celiac disease (n = 11)

Age (years)	Sex	Race	Origin	Digestive Symptoms	Nutritional Status	t T1DM (months)	HbA1c	Immuno histochemistry	Marsh	HLA	Dx
16	F	Mixed	Urban	Diarrhea	Normal	41	8.2	Positive	II	DQB1* 02:01	CD
				Flatulence						DQB1* 03:02*(08)	
				Distension							
				Vomiting							
11	M	Afro	Rural	No	Normal	35	11.0	Positive	II	DQB1* 02:02	CD
										DQB1* 03:02*(08)	
9	F	White	Urban	No	Overweight	61	12.0	Negative	0	DQB1* 02:01	pCD
										DQB1* 03:02*(08)	
9	M	White	Urban	Vomiting	Overweight	60	9.7	Negative	0	DQB1* 02:01	pCD
										DQB1* 02:02	
5	F	White	Rural	Abdominal pain	Normal	18	8.3	Negative	0	DQB1* 02:01	pCD
	
10	M	Mixed	Urban	Abdominal pain	Normal	<1	9.2	Positive	II	DQB1* 02:01	CD
				Vomiting						DQB1* 03:02*(08)	
				Constipation							
14	F	White	Urban	Diarrhea	Obese	140	11.0	Negative	0	DQB1* 02:01	pCD
				Vomiting						DQB1* 03:02*(08)	
				Constipation							
7	F	White	Urban	No	Normal	44	5.6	Negative	0	DQB1* 02:02	pCD
										DQB1* 03:02*(08)	
9	M	Afro	Rural	No	Normal	17	9.2	Negative	0	DQB1* 02:01	pCD
										DQB1* 03:02*(08)	
6	F	White	Urban	Diarrhea	Normal	26	8.1	Negative	0	DQB1* 02:01	pCD
				Flatulence						DQB1* 03:02*(08)	
				Abdominal pain							
16	F	Afro	Urban	Steatorrhea	Normal	44	9.0	Positive	II	DQB1* 02:02	CD
				Distension						DQB1* 03:02*(08)	
				Abdominal pain							
				Weightloss							
				Constipation							

Sx=symptoms, Dx=diagnosis, HLA=histocompatibility antigen, F=female, M=male

CD=celiac disease (positive immunochemistry, Marsh II/III and/or present HLA DQ2/DQ8), pCD=potential CD (negative immunohistochemistry, Marsh 0/I and/or present HLA DQ2/DQ8), t=duration

The comparison between children with T1DM diagnosed with CD and those with T1DM diagnosed as having potential CD is shown in Table 3. The time of presentation of the last ketoacidotic episode after diagnosis of T1DM and CD was significantly higher in children with potential CD than in children with CD (*p* <0.0260). There was a higher risk of presenting with CD (n= 4) in children with T1DM who were between 13 and 18 years of age, of the male sex and with digestive symptoms (constipation, vomiting, distension, steatorrhea, diarrhea, abdominal pain and flatulence), but these findings were not statistically significant (*p* >0.05). The risk of presenting with potential CD (n= 7) was highest for children with T1DM between the ages of 2 and 5 years old who were malnourished, had altered glycosylated hemoglobin and glycemia levels, and had digestive symptoms such as diarrhea, vomiting, flatulence and abdominal pain, but these findings were not statistically significant (*p* >0.05).

**Table 3:** Characteristics of children with T1DM, celiac disease and potential celiac disease. (n=11)

Variables	tTG2	
	CD (n=4)	Potential CD (n=7)
Sociodemographic		
Age (years) X (range)	13 (10-16)	8 (5-14)
Sex (female:male)	2:2	5:2
Origin (urban:rural)	3:1	5:2
Race (white:other)	0:4	6:1
**Antecedents**	** **	** **
Family members with metabolic diseases (n, %)	4 (100.0)	5 (71.4)
**Evolution of the disease**	** **	** **
Duration (months) X (range)	30.0 (0-44)	53.6 (17-140)
No. ketoacidotic comas X (range)	1 (0-2)	0,6 (0-3)
Last ketoacidotic coma (months) X (range)	21.5 (0-43)	69.5 (42-97)
**Nutritional status according to WHO**		
Normal:Malnutrition	4:0	4:3
Normal:Altered height	4:0	7:0
**Paraclinics**	** **	** **
Hemoglobin (gr/dL) X (range)	14.3 (12.9-15.8)	13.3 (12.1-14.5)
Glycosylated hemoglobin (%) X (range)	9.4 (8.2-11.0)	9.1 (5.6-12.0)
Glycemia (gr/dL) X (range)	145.5 (57-246)	228.8 (64.0-560.0)

X=mean; n=number

## Discussion

### Prevalence and seroprevalence

Based on the histopathological findings that 7.1% of children with T1Dm have CD or potential CD, the prevalence in Colombia is higher than that in other Latin American countries such as Brazil (2.6% -4.8%) ^22^ ^-^ ^24^ and Venezuela (1.7%) ^25^. It is also higher than that in Asia ^26^, Europe ^27^ and North America ^27^; but very similar to that of Oceania ^27^, and lower than that of Africa (3.0-11.0%) ^28^ ^,^ ^29^.

The seroprevalence for CD was determined in this study by testing for tTG2, which has a reported sensitivity/specificity of 97.0% ^16^ ^,^ ^17^. This finding allowed the identification of CD in 8.4% of participants; this rate is lower than that reported in Asian, European and other South American countries (11.3%) ^26^ ^,^ ^30^ ^,^ ^31^.

The differing results of these prevalences and seroprevalence rates may be due, among others factors, to genetic and regional characteristics; but primarily to the different antibodies used for CD screening; Therefore, in order to unify and standardize the study of CD, it is suggested that researchers rely on the current algorithms proposed by the ESPGHAN guidelines for the diagnosis of asymptomatic children with a high risk of CD. These guidelines recommend the use of anti-transglutaminase antibodies (tTG2) and/or anti-endomysium (EMA) ^1^.

### Possible associations

Among children with T1DM, there are various factors that increase their risk of presenting with CD. Those risk factors include sex ^32^, age ^32^ ^-^ ^34^, and the presence of thyroid disease ^32^ ^,^ ^34^ ^,^ ^35^; and of greater symptoms ^33^ ^,^ ^36^ decreased weight and height ^34^ ^-^ ^36^, anemia ^35^ ^,^ ^37^, hypoalbuminemia ^35^, rickets ^37^, and hypophosphatemia ^37^. Similar to other authors ^34^ ^,^ ^38^, we found significant differences related to the time of T1DM, specifically in the time (months) of the presentation of the last ketoacidotic coma between children with potential CD and without CD (69.5 ±38.9 months versus 19.1 ±30.7 months, *p*= 0.0260).

This variety of risk factors may depend on sample size, genetics, geographic area, and the specific antibodies used for CD screening, and they should be the focus of future studies, looking for other possible risk factors such as the environmental factors related to breastfeeding, the amount of gluten ingested and the age of introduction in complementary feeding which recently have begun to show controversial results ^39^ ^,^ ^40^.

### Genotype

The most frequent allele of the 11 children with T1DM and CD in our study was DQ2/DQ8. This result is different from those reported in Africa ^28^, Europe ^41^ ^,^ ^42^ and Oceania ^43^, where DQ2 predominates. It is also different from Asia ^44^ and the United States ^45^, where DQ8 is the predominant one. The consulted Latin American studies do not report the alleles ^22^ ^-^ ^25^.

Given that 40.0% of the general population carries HLA-DQ2 or DQ8 and that the risk of presenting in the following 10 years with CD or autoimmunity for CD is increased in these patients ^45^, periodic monitoring is necessary in children with T1DM. Monitoring should be done with serological markers, corroboration with endoscopy and by HLA measurement. This concept makes sense, especially given that HLA DQ2 and DQ8 haplotypes are found in almost all children with CD and are essential for the recognition of gliadin epitopes by antigen-presenting cells. Additionally, if a child is negative for both types of HLA DQ, it is very unlikely that he will have CD, since the negative predictive value is more than 99% ^46^.

Children with a diagnosis of CD, including potential CD, were referred to a pediatric nutritionist, who initiated the nutritional recommendations of a gluten-free diet. Additionally, their parents and siblings (the first-degree relatives) were screened for CD; and together with the children with negative screening results, they will be monitored every six months/annually for CD.

The strengths of the study include that all participants belong to the same cohort of children and were seen by the same healthcare professionals (endocrinologist, gastroenterologist and pathologist) for several years of follow-up (in-hospital cohort). Among the limitations of the study, it is noted that the sample size was limited. Although the population of a tertiary care hospital is described, where a large number of children from southwestern Colombia attend, the results cannot be generalized to all of Colombia, or even to the whole city. In the same way, other possible risk factors such as quality of life, psychological, social, nutritional, and environmental, among others, that could explain the multifactorial model of this entity were not evaluated. Finally, our data were obtained in an intrahospital environment, which allows for some degree of bias.

## Conclusion

The prevalence of CD in these children with T1DM is higher than that reported in other South American countries. The prevalence of CD was found to be associated with the time of presentation of T1DM and its main allele, the DQ2/DQ8. These findings are different from what has been described in other places around the world.
