# Supplementary material for: Prevalence of markers of celiac disease in Colombian children with diabetes mellitus type 1
Source: Colomb Med (Cali). 2018 Dec 30;49(4):273–9. doi: 10.25100/cm.v49i3.3650 (PMC6342086; doi:10.25100/cm.v49i3.3650)
Supplement: Related texts

# Original article: Prevalencia de marcadores de enfermedad celíaca en niños Colombianos con diabetes mellitus tipo 1

## Introducción

La enfermedad celíaca (EC) es un trastorno sistémico mediado por el sistema inmune, provocado por el gluten y las prolaminas relacionadas, en individuos genéticamente susceptibles y se caracteriza por la presencia de una combinación variable de manifestaciones clínicas dependientes del gluten, anticuerpos específicos para EC, haplotipos HLA-DQ2 o HLA-DQ8 y enteropatía ^1^.

La asociación entre la diabetes mellitus tipo 1 (DMT1) y la EC está bien establecida; sin embargo, dentro de los últimos años, varios estudios han demostrado que la prevalencia de EC en pacientes diabéticos es aún mayor de lo considerado anteriormente ^2^.

Las Guías para el diagnóstico de EC de la Sociedad Europea de Gastroenterología, Hepatología y Nutrición Pediátrica ESPGHAN sugieren realizar pruebas a los niños asintomáticos con riesgo genético para desarrollar EC, como Diabetes mellitus tipo 1 (DMT1) ^1^. 

La prevalencia más alta reportada a nivel mundial de EC es en El Sahara, África (5.6%); seguida en Oceanía (1.2%); Europa (1.0%); Estados Unidos (0.8%); Asia (0.3%-0.7%), y Brasil y Argentina (0.14%-0.60%) ^3^; siendo esta prevalencia mayor en pacientes con DMT1 (2.4%-16.4%) ^4^. En una revisión sistemática y de meta-regresión realizada en Colombia, se concluye que la EC parece ser una condición rara en los colombianos ^5^. A pesar que es bien conocido a nivel mundial, que hay mayor prevalencia de EC en niños con DMT1 en comparación con la población general ^6^, y que la mayoría de estos niños son asintomáticos al momento del diagnóstico ^7^^,^^8^; aún nos falta en Colombia, entre otras, resolver varias preguntas de investigación, tales como si este riesgo es mayor en los menores de 5 años como lo han demostrado los europeos ^9^, o con predominio del género femenino como en la población general adulta ^10^ o sobre los datos controversiales de la coexistencia de EC y DMT1 y el control glucémico ^11^^-^^14^ e incluso reportar nuestra prevalencia, características clínicas y paraclínicas de niños con EC y DMT1 coexistentes; por lo que se analizaron las bases de datos del Servicio de Endocrinología Pediátrica del Hospital Universitario del Valle “Evaristo García” de Cali, Colombia de los niños con diagnóstico de DMT1 con el objetivo de determinar la prevalencia de marcadores de EC en un grupo de niños con DMT1, analizando las manifestaciones clínicas, inmunológicas y genéticas.

## Materiales y Métodos

Se diagnosticó DMT1 por destrucción de las células beta en todos los niños mayores de 6 meses de edad sin importar si debuta o no en cetoacidosis, haya o no presencia de otras enfermedades autoinmunes; y en mayores de 10 años si están obesos, de igual manera, hasta que se demuestre ausencia de autoinmunidad ^15^. Se diagnosticó EC cuando la IgA anti-transglutaminasa (tTG2) fue positiva; el haplotipo HLA DQ2 y HLA DQ8 compatible; la histotología del duodeno demostró anormalidad de la vellosidad intestinal con un Marsh grado II o mas y se presentó una combinación variable de manifestaciones clínicas dependientes del gluten; y EC potencial cuando la tTG2 fue positiva, el HLA compatible y sin anomalías histológicas a la biopsia duodenal, con o sin signos y síntomas ^1^.

Se realizó un estudio observacional descriptivo de prevalencia en niños con diagnóstico de DMT1 que consultaron entre el 2 de agosto de 2013 y el 23 de febrero de 2017 a la Consulta de Endocrinología Pediátrica del Hospital Universitario del Valle “Evaristo García” de Cali, Colombia, institución de tercer nivel de atención del suroccidente del país. Los criterios de inclusión fueron niños con diagnóstico de DMT1, de ambos sexos, mayores de 6 meses de edad que en su alimentación complementaria ya habían consumido gluten; y los criterios de exclusión fueron niños con diagnóstico de diabetes mellitus congénita, en coma cetoacidótico, con cromosomopatías asociadas como Síndrome de Down, con otras enfermedades autoinmunes asociadas como hipotiroidismo, con diagnóstico previo de EC, y con enfermedad inflamatoria intestinal. Se tuvieron en cuenta variables sociodemográficas como edad, sexo, raza y orígen; antrompométricas como peso y talla; de la enfermedad como duración de la DMT1, número de comas cetoacidóticos y fecha de último coma cetoacidótico; paraclínicas como hemoglobina glicosilada, hemoglobina y glicemia, y familiares metabólicas como hipertensión, diabetes, sobrepeso y obesidad. Las variables clínicas digestivas que se tuvieron en cuenta fueron el estreñimiento, el vómito, la distensión abdominal, la esteatorrea, la diarrea, el dolor abdominal, las flatulencias y la pérdida de peso. Se realizó tamizaje mediante una Ig A anti-transglutaminasa (tTG2) Biocard^TM^ Celiac Test Ani Biotech, Vantaa, Finland (97.4% sensibilidad, 96.9% especificidad) ^16^^,^^17^. A los niños tTG2 positivos, se les realizó toma de mínimo 4 biopsias del bulbo y segunda porción duodenal. La evaluación del material de biopsia fue realizado por el mismo patólogo luego de la tinción de hematoxilina-eosina. La inmunohistoquímica del antígeno leucocito comun fue usada para la evaluación del conteo de leucocitos intraepiteliales, y la presencia de mas de 30 linfocitos intraepiteliales versus 100 células epiteliales detectados por antígeno leucocito comun fue considerado como linfocitosis intraepitelial ^18^^,^^19^. Según Marsh-Oberhuber la histopatología fue clasificada como Marsh I cuando se demostró “lesión infiltrativa”, “lesión infiltrativa-hiperplásica” como Marsh II, y “atrofia vellosa” como Marsh III (parcial IIIa, subtotal IIIb y total IIIc) ^20^. A estos niños se les realizó genotipificación del HLA DQ2 y HLA DQ8, por reacción en cadena de polimerasa ^21^. 

La aceptación de participar en el estudio se logra mediante la firma del consentimiento informado de los padres o tutores y el asentimiento informado de los niños mayores de 7 años, siendo este trabajo aprobado por el Comité de Ética de la Universidad del Valle y del Hospital Universitario del Valle “Evaristo García” de Cali, Colombia.

El tamaño de muestra fue por conveniencia, participando todos los niños con diagnóstico de DMT1 que cumplieron con los criterios de inclusión del estudio, aceptaron participar en el estudio y asistieron a la Consulta de Endocrinología Pediátrica del Hospital Universitario del Valle “Evaristo García” de Cali, Colombia. Se estimó de la proporción de niños con DMT1 y EC y su correspondiente intervalo de confianza al 95%; en el total de niños participantes se estimaron porcentajes, percentiles, promedios, medianas y las demás medidas descriptivas con sus correspondientes desviaciones estándar y rangos, y para evaluar las posibles asociaciones, se realizó inicialmente análisis univariado entre cada una de las variables de exposición de interés y la variable efecto. Además, se exploró la posible ocurrencia asociación entre las variables de exposición de mayor interés y otras covariables, y entre la variable resultado de interés (EC) y las demás covariables, con el objetivo de evaluar la posible existencia de confusión. Para ello, se construyeron gráficos y tablas de 2x2 y se estimaron los ORs con sus respectivos intervalos de confianza (95%). Para valorar la significancia estadística se utilizó la prueba exacta de Fisher y un valor de *p* <0.05, a dos colas, se consideró como estadísticamente significativo.

## Resultados

Fueron incluidos 155 niños, 83 del sexo masculino, con edad promedio de 11 años (2-18), con diagnóstico de DMT1, quienes tuvieron una duración promedio de la enfermedad de 46.1 meses (0-180 meses), con promedio de un episodio de coma cetoacidótico (0-7 episodios) y con presencia del último episodio cetoacidótico promedio de 20.5 meses (0-163 meses). La mayoría (73.6%; 114/155) presentaron algún antecedente familiar con problemas metabólicos. Los paraclínicos fueron: hemoglobina glicosilada de 9.1% (5.2-19.0%), glicemia de 193.1 g/dL (36.1-600.0 g/dL) y hemoglobina de 12.9 g/dL (9.1-15.0 g/dL). La mayoría de ellos provenían de ciudad urbana (n=109, 70.3%). La tTG2 fue positiva en el 8.4% (13/155). Los parámetros antropométricos se muestran en la Tabla 1. 

**Tabla 1:** Características de los niños con Diabetes mellitus tipo 1 e Ig A anti-transglutaminasa(n= 155)

Variables	tTG2	
	Positiva (n= 13 )	Negativa (n= 142)
Sociodemográficas	8.4% (IC 95% 6.2-10.6)	91.6% (IC 95% 89.3-93.8)
Edad (años) X (rango)	10 (5-16)	11 (2-18)
Sexo femenino:masculino	7:6	76:66
Origen urbano:rural	10:3	99:43
Raza blanca:otra	6:7	73:69
**Antecedentes**	** **	** **
Familiares metabólicos presentes n, %	10 (76.9)	104 (73.2)
**Evolución de la enfermedad**	** **	** **
Duración (meses) X (rango)	38.1 (0-140)	46.8 (0-180)
No. comas cetoacidóticos X (rango)	1 (0-3)	1 (0-7)
Último coma cetoacidótico (meses) X (rango)	36.4 (0-97)	19.4 (0-163)
**Estado nutricional según OMS**		
Normal:Malnutrición	8:5	102:40
Normal:Talla alterada	13:0	126:16
**Paraclínicos**	** **	** **
Hemoglobina (gr/dl) X (rango)	13.2 (11.0-15.8)	12.9 (9.1-15.8)
Hemoglobina glicosilada (%) X (rango)	9.3 (5.6-12.0)	9.1 (5.2-9.0)
Glicemia (gr/dl) X (rango)	179.2 (57-560)	194.4 (36.1-600.0)

X=promedio; n=número

De los 155 niños incluidos, 13 tuvieron tTG2 positiva (8.4%), y a once de ellos, se les realizó endoscopia de vías digestivas altas con toma de biopsias intestinales y haplotipos HLA DQ2 y HLA DQ8 (Figura 1). Los 4 niños con EC por inmunohistoquímica positiva, presentaron en los anticuerpos CD3, CD8 y CD45 comparados con testigos adecuados, un número y localización concordante con EC. En la Tabla 2, se describen las características generales de los 11 niños con DMT1, EC potencial (n= 7) y EC (n= 4). 

**Figura 1:** Flujograma para el estudio de niños con DMT1 con EC

**Tabla 2:** Características generales en niños con Diabetes mellitus tipo 1 y Enfermedad celíaca (n=11)

Edad (años)	Sexo	Raza	Origen	Síntomas Digestivos	Diagnóstico Nutricional	t DMT1 (meses)	HbA1c	Inmuno Histoquímica	Marsh	HLA	Dx
16	F	Mestiza	Urbano	Diarrea	Eutrófico	41	8.2	Positiva	II	DQB1* 02:01	EC
				Flatos						DQB1* 03:02*(08)	
				Distensión							
				Vómito							
11	M	Afro	Rural	No	Eutrófico	35	11.0	Positiva	II	DQB1* 02:02	EC
										DQB1* 03:02*(08)	
9	F	Blanca	Urbano	No	Sobrepeso	61	12.0	Negativa	0	DQB1* 02:01	Ecp
										DQB1* 03:02*(08)	
9	M	Blanca	Urbano	Vómito	Sobrepeso	60	9.7	Negativa	0	DQB1* 02:01	ECp
										DQB1* 02:02	
5	F	Blanca	Rural	Dolor abdominal	Eutrófico	18	8.3	Negativa	0	DQB1* 02:01	ECp
	
10	M	Mestiza	Urbano	Dolor abdominal	Eutrófico	<1	9.2	Positiva	II	DQB1* 02:01	EC
				Vómito						DQB1* 03:02*(08)	
				Estreñimiento							
14	F	Blanca	Urbano	Diarrea	Obeso	140	11.0	Negativa	0	DQB1* 02:01	ECp
				Vómito						DQB1* 03:02*(08)	
				Estreñimiento							
7	F	Blanca	Urbano	No	Eutrófica	44	5.6	Negativa	0	DQB1* 02:02	ECp
										DQB1* 03:02*(08)	
9	M	Afro	Rural	No	Eutrófico	17	9.2	Negativa	0	DQB1* 02:01	ECp
										DQB1* 03:02*(08)	
6	F	Blanca	Urbano	Diarrea	Eutrófico	26	8.1	Negativa	0	DQB1* 02:01	ECp
				Flatos						DQB1* 03:02*(08)	
				Dolor abdominal							
16	F	Afro	Urbano	Esteatorrea	Eutrófico	44	9.0	Positiva	II	DQB1* 02:02	EC
				Distensión						DQB1* 03:02*(08)	
				Dolor abdominal							
				Pérdida de peso							
				Estreñimiento							

Sx=síntomas, Dx=diagnóstico, HLA=antígeno de histocompatibilidad, F=femenino, M=masculino

EC=enfermedad celíaca (inmunohistoquímica positiva, Marsh II/III y/o HLA DQ2/DQ8 presente), Ecp=EC potencial (inmunohistoquímica negativa, Marsh 0/I y/o HLA DQ2/DQ8 presente), t=duración

La comparación entre los niños con DMT1 diagnosticados como EC y con DMT1 diagnosticados como EC potencial se muestran en la Tabla 3. El tiempo de presentación del último episodio cetoacidótico luego del diagnóstico de DMT1 y EC, fue significativamente mayor en los niños con EC potencial que en los niños con EC (*p* < 0.0260). Tuvieron mayor riesgo de presentar EC (n= 4) los niños con DMT1 entre los 13 y 18 años edad, del género masculino y con síntomas digestivos (estreñimiento, vomito, distensión, esteatorrea, diarrea, dolor abdominal y flatulencias), pero sin significancia estadística (*p* >0.05); y de presentar EC potencial (n= 7) los niños con DMT1 entre los 2 y 5 años, malnutridos, con hemoglobina glicosilada y glicemia alteradas, y con síntomas digestivos como diarrea, vomito, flatulencias y dolor abdominal, pero sin diferencias estadísticas (*p* >0.05). 

**Tabla 3:** Características de los niños con DMT1, Enfermedad celíaca y Enfermedad celíaca potencial. (n= 11)

Variables	tTG2	
	EC (n= 4)	EC potencial (n= 7)
sociodemográficas		
Edad (años) X (rango)	13 (10-16)	8 (5-14)
Sexo femenino:masculino	2:2	5:2
Origen urbano:rural	3:1	5:2
Raza blanca:otra	0:4	6:1
**Antecedentes**	** **	** **
Familiares metabólicos presentes n, %	4 (100.0)	5 (71.4)
**Evolución de la enfermedad**	** **	** **
Duración (meses) X (rango)	30.0 (0.0-44.0)	53.6 (17.0-140.)
No. comas cetoacidóticos X (rango)	1.0 (0.0-2.0)	0.6 (0.0-3.0)
Último coma cetoacidótico (meses) X (rango)	21.5 (0.0-43.0)	69.5 (42.0-97.0)
**Estado nutricional según OMS**		
Normal:Malnutrición	4:0	4:3
Normal:Talla alterada	4:0	7:0
**Paraclínicos**	** **	** **
Hemoglobina (g/dL) X (rango)	14.3 (12.9-15.8)	13.3 (12.1-14.5)
Hemoglobina glicosilada (%) X (rango)	9.4 (8.2-11.0)	9.1 (5.6-12.0)
Glicemia (g/dL) X (rango)	145.5 (57-246)	228.8 (64.0-560.0)

X=promedio; n=número

## Discusión

### Prevalencia y seroprevalencia

Nuestra prevalencia basada en los hallazgos histopatológicos del 7,1% de EC, incluida la EC potencial, en niños con DMT1, es superior a la de otros países latinoamericanos como Brasil (2.6%-4.8%) ^22^^-^^24^ y Venezuela (1.7%) ^25^; así como a la de Asia ^26^, Europa ^27^ y Norteamérica ^27^; pero muy similar a la de Oceanía ^(279^, e inferior a la de África (3.0-11.0%) ^28^^,^^29^. 

Con relación a la seroprevalencia para EC en este estudio, el tamizaje se llevó a cabo con la tTG2, cuya sensibilidad/especificidad reportada del 97.0% ^16^^,^^17^; permitió identificarla en un 8.4%; inferior a lo reportado en otros países asiáticos, europeos y suramericanos, que es del 11.3% ^26^^,^^30^^,^^31^.

Los resultados variables y diversos de estas prevalencias y seroprevalencias, se pueden deber, entre otras, a características genéticas y regionales; y principalmente a los diferentes anticuerpos utilizados para el tamizaje de EC; por lo que para unificar y estandarizar conceptos, se sugiere basarse en los actuales algoritmos propuestos por las Guías de la ESPGHAN para el diagnóstico de niños asintomáticos con riesgo elevado de EC, que recomiendan el uso de anticuerpos anti-transglutaminasa (tTG2) y/o anti-endomisio (EMA) ^1^.

### Posibles asociaciones

Entre los diversos factores de riesgo en niños con DMT1 para presentar EC están el sexo ^32^, la edad ^32^^-^^34^, la presencia de enfermedad tiroidea ^32^^,^^34^^,^^35^; y de mayores síntomas ^33^^,^^36^ (bajo peso y talla ^34^^-^^36^, anemia ^35^^,^^37^, hipoalbuminemia ^35^; raquitismo ^37^, hipofosfatemia ^37^. Al igual que otros autores ^34^^,^^38^, nosotros encontramos diferencias significativas relacionadas con el tiempo de la DMT1, específicamente en el tiempo (meses) de la presentación del último coma cetoacidótico entre los niños con EC potencial y sin EC (69.5 ±38.9 meses versus 19.1 ±30.7 meses, *p*= 0.0260).

Esta diversidad de factores de riesgo, dependientes del tamaño de muestra, de la genética, del área geográfica, de los anticuerpos utilizados para el tamizaje de EC; deberán ir enfocados a futuro, a la búsqueda de otros posibles factores de riesgo como los ambientales relacionados con lactancia materna, cantidad de gluten ingerido y edad de introducción en la alimentación complementaria; los cuales, al día de hoy comienzan a mostrar resultados controversiales ^39^^,^^40^.

### Genotipo

El alelo más frecuente de los 11 niños con DMT1 y EC de nuestro estudio, fue el DQ2/DQ8, datos diferentes a los reportados en África ^28^, Europa ^41^^,^^42^ y Oceanía ^43^, donde predomina el DQ2 y a los descritos en Asia ^44^ y Estados Unidos ^45^, donde el DQ8 es el predominante. Los estudios Latinoamericanos consultados no informan los alelos ^22^^-^^25^. 

Sabiendo que el 40.0% de la población general porta HLA-DQ2 o DQ8, y el riesgo a presentar en los siguientes 10 años EC o autoinmunidad para EC ^45^, es necesario el control periódico en los niños con DMT1 con marcadores serológicos, corroboración endoscópica y medición de HLA. Este concepto es mas que válido, teniendo en cuenta que los haplotipos HLA DQ2 y DQ8 se encuentran en casi todos los niños con EC, que son esenciales para el reconocimiento de epítopes de gliadina por las células presentadoras de antígenos; y que si un niño es negativo para ambos tipos de HLA DQ, es muy poco probable que presente EC, ya que el valor predictivo negativo es más del 99% ^46^.

Los niños con diagnóstico de EC, incluida la EC potencial, fueron remitidos a la nutricionista infantil, quien da inicio a las recomendaciones nutricionales sobre dieta libre en glúten, y a sus padres y hermanos (primera línea de consanguinidad) se les inicia el tamizaje para EC; y junto con los niños con tamizaje negativo, se les continua monitorización semestral/anual para EC. 

Las fortalezas del estudio incluyen que todos los participantes pertenecen a una misma cohorte de niños que son vistos por los mismos profesionales de la salud (endocrinólogo, gastroenterólogo y patólogo) durante varios años de seguimiento (cohorte intrahospitalaria). Entre las limitaciones del estudio; a pesar que se describe la población de un hospital de tercer nivel de atención donde asiste una gran cantidad de niños del suroccidente colombiano, el tamaño de la muestra puede considerarse limitado, y así, los resultados no puedan ser generalizados a toda Colombia, ni siquiera a toda la ciudad. De la misma manera, no se preguntaron otros posibles factores de riesgo como calidad de vida, sicológicos, sociales, nutricionales, y ambientales, entre otros, que puedan explicar el modelo multifactorial de esta entidad. Finalmente, nuestros datos fueron obtenidos en un ambiente intrahospitalario, lo que permite algún grado de sesgo.

## Conclusión

La prevalencia de EC en estos niños con DMT1 es superior a la de otros países suramericanos; estando asociada al tiempo de presentación de la DMT1 y su principal alelo el DQ2/DQ8, hallazgos diferentes a lo descrito a nivel mundial.
